# Modification of Electrode Interface with Fullerene-Based Self-Assembled Monolayer for High-Performance Organic Optoelectronic Devices

**DOI:** 10.3390/mi13101613

**Published:** 2022-09-27

**Authors:** Dong Hun Sin, Soo Hyun Kim, Jaewon Lee, Hansol Lee

**Affiliations:** 1 Department of Chemical Engineering, Pohang University of Science and Technology, Pohang 37673, Korea; 2 Department of Chemical and Biological Engineering, Gachon University, Seongnam 13120, Korea; 3 Department of Chemical Engineering and Applied Chemistry, Chungnam National University, Daejeon 34134, Korea

**Keywords:** organic optoelectronic devices, organic solar cells, cathode interlayer, self-assembled monolayer, fullerene

## Abstract

Efficient charge transfer between organic semiconductors and electrode materials at electrode interfaces is essential for achieving high-performance organic optoelectronic devices. For efficient charge injection and extraction at the electrode interface, an interlayer is usually introduced between the organic active layer and electrode. Here, a simple and effective approach for further improving charge transfer at the organic active layer–interlayer interface was presented. Treatment of the zinc oxide (ZnO) interlayer, a commonly used n-type interlayer, with a fullerene-based self-assembled monolayer (SAM) effectively improved electron transfer at the organic–ZnO interface, without affecting the morphology and crystalline structure of the organic active layer on the cathode interlayer. Furthermore, this treatment reduced charge recombination in the device, attributed to the improved charge extraction and reduction of undesirable ZnO-donor polymer contacts. The photocurrent density and power conversion efficiency of organic solar cells employing the fullerene-SAM-treated interlayer were ~10% higher than those of the device employing the nontreated interlayer. This improvement arises from the enhanced electron extraction and reduced charge recombination.

## 1. Introduction

Organic electronic devices, in which organic semiconductor materials are the active materials, are among the most promising devices for the realization of future wearable electronics because of the inherent high mechanical flexibility and easy processability of organic materials [1,2,3,4,5,6,7]. To achieve high-performance organic electronic devices, it is essential to achieve proper electrical contact between the organic semiconductor and electrode materials [8,9,10,11,12,13]. In most cases, efficient charge transfer at the organic–electrode interface is required to ensure efficient charge injection and extraction, and high charge carrier selectivity is essential for preventing unwanted transfer of opposite charge carriers [14,15]. An effective way to realize such an interface is by inserting an interlayer between the organic layer and electrode. Numerous studies have been conducted to develop interlayer materials and control their properties [10,14,15,16,17].

Organic solar cells are one of the most representative organic electronic devices, where the importance of the interlayers is well-reflected. The cathode and anode of organic solar cells should efficiently extract photogenerated electrons and holes from the organic photoactive layer, respectively [14]. Most high-performance organic solar cells use a bulk heterojunction comprising donor and acceptor materials as the photoactive layer, and that the undesirable electrical contacts between the organic and electrode materials are inevitable in such structures (i.e., contact between the donor and cathode or contact between the acceptor and anode) [18]. Therefore, the organic–electrode interface should exhibit charge carrier selectivity to prevent the injection of charge carriers into the wrong electrode [15]. Cathode and anode interlayers perform such functions, and, thus, the performance of organic solar cells greatly depends on the properties of the interlayers [10,17].

Surface treatment of the interlayers can further improve the electrical properties of the organic–electrode interface. A representative approach is the formation of a self-assembled monolayer (SAM) on the interlayer surface. SAMs are two-dimensional molecular arrangements that spontaneously form on the surface of a substrate [19,20]. The head group of a SAM forms a covalent bond with the surface of the target substrate, where the surface properties of the substrate can greatly vary depending on the end-group and molecular order of the SAM [20,21,22,23]. For example, surface properties such as the surface energy, surface roughness, and formation of surface dipoles can be controlled by SAM treatment [21,24,25,26]. Such modifications of the substrate surface or interlayers lead to changes in the microstructure and energy level alignment of the organic semiconductors deposited on the SAM-treated surface, which can prospectively improve the performance of organic electronic devices [20,24,25,26]. Interlayers with modified surface characteristics, achieved through SAM treatment, have also been applied to organic solar cells (Table 1), where prior studies generally focused on lowering the energy barrier at the organic–electrode interface by controlling the energy level alignment at the interface via SAM treatment [23,27,28,29].

In previous studies, SAMs with fullerene-based end-groups (fullerene-based SAMs) were used to modify the cathode interlayer and enhance the performance of organic solar cells [30,31]. Fullerene, a well-known electron acceptor commonly used in organic solar cells, was expected to facilitate electron transfer to the interlayer and cathode when positioned on the cathode interlayer as the end-group of the SAM. Indeed, it has been successfully demonstrated that the power conversion efficiency (PCE) and environmental stability of organic solar cells are increased by the introduction of fullerene-based SAMs on metal oxide cathode interlayers, such as zinc oxide (ZnO) and titanium oxide (TiO_x_) [31,32,33,34,35,36,37,38]. Previous studies attributed the enhancements to the passivation of surface traps in the interlayers and reduced charge recombination [35,39]. However, most of these conclusions are simply based on the change in the current density–voltage (*J*–*V*) characteristics of the devices and thus lack direct observations of the charge carrier dynamics and deep understanding of the role of the SAM in improving the device performance.

In the present study, we demonstrated the effectiveness of fullerene-SAM treatment of the cathode interlayer surface for improving the extraction of electrons from organic semiconductors to the cathode, together with detailed investigations of the changes in the charge carrier dynamics induced by SAM treatment. A fullerene-based SAM is formed on the ZnO surface by simple spin-casting of the precursor solution. The SAM does not affect the microstructure of the organic semiconductor layer deposited on it, preventing unwanted changes in the properties of the organic semiconductors. When applied to organic solar cells, introduction of the fullerene-based SAM accelerates the extraction of photogenerated electrons compared with the cathode interface without SAM treatment. The charge carrier lifetime is also increased by SAM treatment, leading to reduced charge recombination within the device. The reduced charge recombination is attributed to not only the improved charge extraction but also the elimination of undesirable contact between the cathode and donor polymer. Finally, these changes result in an increase in the photocurrent density and PCE of solar cells, where the values are ~10% higher than those of the devices without SAM treatment.

## 2. Materials and Methods

### 2.1. Materials

The conjugated polymer P4TNTz-2F was synthesized by following the procedure reported in a previous study and was used as the donor polymer, consisting of a photoactive layer [40]. [6,6]-Phenyl-C71-butyric acid methyl ester (PC_71_BM) was purchased and used as the acceptor for the photoactive layer without further purification (Nano-C, Westwood, MA, USA). The fullerene-based SAM used in this study, NPC70-OH, was synthesized by following a previously published procedure [39]. The ZnO nanoparticle solution was prepared by following a previously reported method and was spin-coated onto the substrate to obtain a ZnO cathode interlayer [14,41].

### 2.2. Preparation of Fullerene-SAM-Treated and Nontreated Substrates

Indium tin oxide (ITO) coated glass substrates were sequentially cleaned with detergent, deionized water, acetone, and isopropyl alcohol. The substrates were then completely dried in a convection oven and subjected to UV-ozone treatment for 10 min. The ZnO nanoparticles (15 mg mL^−1^) dispersed in a butanol:methanol mixed solvent (1:1 by volume) were then spin-coated onto the substrates to obtain a ZnO interlayer (~40 nm). For the fullerene-SAM treatment, an NPC70-OH solution (3 mM) in a mixed solvent of tetrahydrofuran (THF):chlorobenzene (CB) (1:1 by volume) was prepared and spin-coated onto the ZnO-coated substrates. Before spin-coating, the solution was stirred for 3 h at room temperature and filtered through a polytetrafluoroethylene (PTFE) filter with 0.2 µm pore size. Spin-coating was performed at 3500 rpm for 1 min. To remove excess unreacted NPC70-OH molecules on the ZnO surface, the substrates were thoroughly rinsed with the THF:CB solvent. Finally, the substrates were stored under vacuum for 12 h and used to fabricate organic solar cells. For nontreated substrates, the ZnO-coated substrates were rinsed with the THF:CB mixed solvent (without NPC70-OH) and then were stored under vacuum for 12 h.

### 2.3. Device Fabrication

A P4TNTz-2F:PC_71_BM (1:1.5 by weight) blend solution (37.7 mg mL^−1^) was prepared using CB as the solvent at 70 °C. Before spin-coating the substrates, 1,8-diiodooctane (DIO) was added to the blended solution. The blended solution was then spin-coated onto the substrate; the spin speed was adjusted to obtain an optimal active layer thickness of ~350 nm. After drying, molybdenum trioxide (MoO_3_, 3 nm) and Au (60 nm) were sequentially deposited on the active layer by thermal evaporation under high vacuum (~10^–7^ Torr) to complete the fabrication of the solar cell.

### 2.4. Characterizations

The *J*–*V* characteristics of the devices were evaluated using a Keithley 4200 power source. AM1.5G solar illumination was simulated using an Oriel Sol^3^A class AAA solar simulator with a 1 kW Xe lamp. The light was referenced to a KG5-filtered Si reference cell (91150V, Newport). Measurements of the devices were performed in a N_2_-filled glove box. The external quantum efficiencies (EQEs) of the devices were measured using an IQE-200 quantum efficiency measurement system (Newport). UV–vis absorption spectra and photoluminescence (PL) spectra were acquired using a CARY-5000 spectrometer (Varian) and an FP-6500 spectrofluorometer (Jasco), respectively. Atomic force microscopy (AFM) images were obtained in tapping mode using a multimode scanning probe microscope (Veeco). Grazing-incidence wide-angle X-ray scattering (GIWAXS) measurements were performed at the 3C and 9A beamlines of the Pohang Accelerator Laboratory (PAL) in Korea. For the measurements, samples were prepared on silicon wafer substrates instead of ITO-coated glass substrates, and the other layers were deposited in the same manner as in the device fabrication. Transient photovoltage (TPV) and transient photocurrent (TPC) decays were measured using a TDC3054C oscilloscope (Tektronix) connected to a high-speed preamplifier (SR560 (Stanford Research Systems) and DHPCA-100 (Femto)). The devices were excited by a 3 ns pulse laser with a wavelength of 532 nm to generate transients under background AM 1.5G illumination at an intensity of 100 mW cm^−2^. The water contact angles on the ZnO surface with and without SAM treatment were measured using a SmartDrop Plus contact angle meter (Femtobiomed).

## 3. Results and Discussion

**Figure 1:**
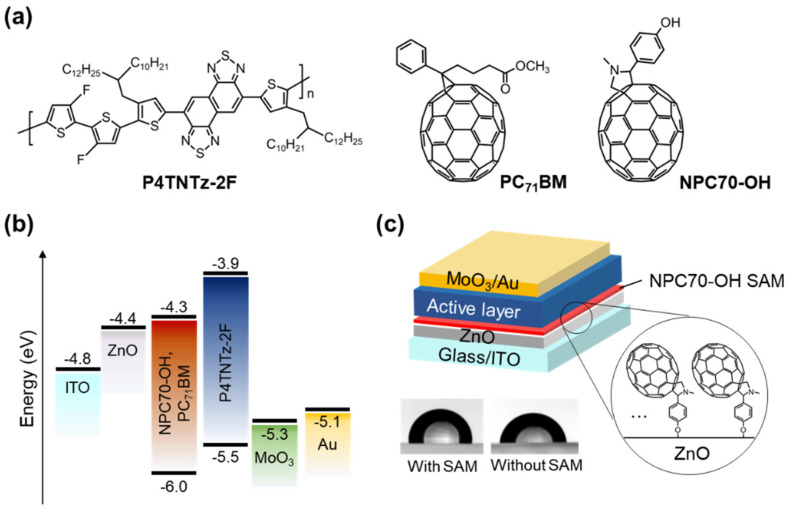
Materials and device structure used in this study. (**a**) Chemical structures of materials. (**b**) Energy level diagram of materials used for device fabrication. (**c**) Device structure of solar cell with fullerene-SAM treatment on ZnO surface. Inset: Water contact angle measurements on ZnO surface with and without fullerene-SAM.

The chemical structures of the materials used in this study are shown in Figure 1a. The donor polymer P4TNTz-2F is a semicrystalline conjugated polymer that has a high PCE of ~10.6% when blended with the acceptor PC_71_BM [40]. According to documented reports, the highest occupied molecular orbital (HOMO) and lowest unoccupied molecular orbital (LUMO) energy levels of P4TNTz-2F are −5.5 and −3.9 eV, respectively, whereas those of PC_71_BM are −4.3 and −6.0 eV, respectively [40]. The LUMO energy level of NPC70-OH is also almost the same at −4.3 eV [39], as can be expected from the structural similarity of PC_71_BM and NPC70-OH. Therefore, separation of the photogenerated excitons at the P4TNTz-2F/PC_71_BM interface and subsequent electron transfer from PC_71_BM to NPC70-OH are considered energetically favorable. NPC70-OH has a –OH head group that can form a covalent bond with the ZnO interlayer. NPC70-OH was selected because of the short length between the head group and C70 fullerene-based end-group, which is beneficial for transferring electrons from the end-group to ZnO interlayer. The energy diagrams of the materials and device structure are illustrated in Figure 1b,c, respectively.

The fullerene-SAM-treated ZnO interlayer was obtained by spin-coating the NPC70-OH solution onto the ZnO surface. The formation of covalent bonds between NPC70-OH and ZnO is evidenced by the increase in the water contact angle on the SAM-treated surface, which was maintained despite thorough rinsing with pure solvents. The water contact angle of the pristine ZnO interlayer surface is 77° and increases to 92° after the fullerene-SAM treatment (Figure 1c, inset). This increase indicates that the surface became more hydrophobic as a consequence of the formation of the fullerene-SAM. The contact angle is similar to or even higher than the previously reported values for fullerene-SAM-treated ZnO surfaces [35], indicating that NPC70-OH effectively formed a SAM on the ZnO layer used in this study.

**Figure 2:**
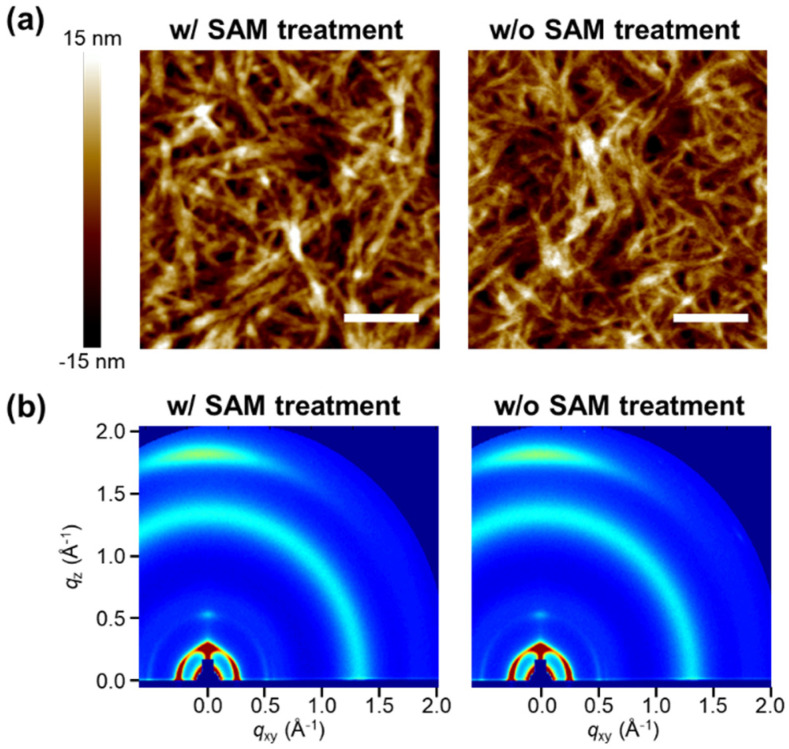
Microstructural characterization of P4TNTz-2F:PC_71_BM blend films. Left: blend film on NPC70-OH/ZnO (with SAM treatment). Right: blend film on ZnO (without SAM treatment). (**a**) AFM height images. Scale bar: 500 nm. Root-mean-square roughnesses are 4.22 nm and 4.16 nm, respectively. (**b**) 2D GIWAXS images.

When the P4TNTz-2F:PC_71_BM blend solution was spin-coated onto the ZnO or NPC70-OH SAM/ZnO surface, the microstructure of the blend film was not affected by the presence of the SAM, resulting in an almost identical crystalline structure and blend morphology (Figure 2). The surface morphologies of the blend films were characterized using AFM (Figure 2a). The blended films show a fibrillar crystalline structure of P4TNTz-2F, as reported in a previous study [40]. The surface morphology and root-mean-square surface roughness are nearly the same (~4.2 nm), regardless of the SAM treatment of the underlying ZnO interlayer. GIWAXS measurements also reveal an almost unchanged crystalline structure of the blend film after the SAM treatment (Figure 2b). The intense (100) diffraction peaks of P4TNTz-2F at *q* = 0.26 Å^−1^ appear along the out-of-plane and in-plane directions, indicating that the molecular orientation of the P4TNTz-2F crystallites is arranged in a bimodal (edge-on and face-on) distribution. The strong (010) diffraction peak at *q* = 1.80 Å^−1^ along the out-of-plane direction confirms the presence of face-on crystallites of P4TNTz-2F. The strong (010) peak is in line with the observations from the AFM images, where the polymer chains are stacked by π–π interactions and form fibrillar crystalline structures. Notably, the microstructure of the blend is not affected by the SAM treatment, as confirmed by the similar diffraction patterns of the blend films on the ZnO and NPC70-OH SAM/ZnO surfaces.

**Figure 3:**
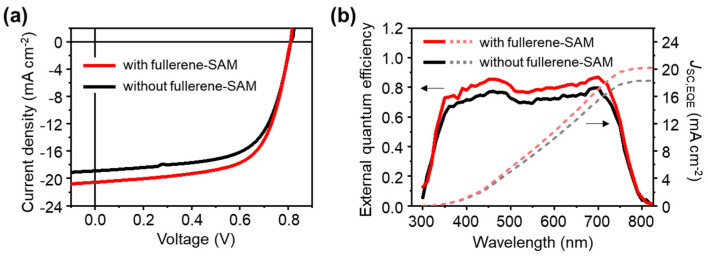
Photovoltaic characteristics of devices with and without fullerene-SAM treatment. (**a**) *J*–*V* characteristics. (**b**) EQE spectra and corresponding *J*_SC,EQE_, calculated by integrating EQE data.

Organic solar cells were fabricated with the ITO/ZnO/P4TNTz-2F:PC71BM/MoO3/Au and ITO/ZnO/NPC70-OH SAM/P4TNTz-2F:PC71BM/MoO3/Au structures, and their photovoltaic properties are compared under AM1.5 G solar illumination at 100 mW cm^−2^ (Figure 3a, Table 2). De-spite the structural similarity of the devices, except for the presence of the fuller-ene-SAM, the J-V characteristics of the devices differ, especially the magnitude of the photocurrent density. The device with the fullerene-SAM-treated ZnO interlayer ex-hibits a higher short-circuit current density (*J*_SC_) than the device with the untreated ZnO interlayer. However, the open-circuit voltage (VOC) and fill factor (FF) of the de-vices are similar. Because of the increased *J*_SC_, the device with the SAM-treated ZnO interlayer exhibits a higher PCE (10.4% in average) than the device with the untreated ZnO interlayer (9.38% in average). The EQE spectra of the devices are also character-ized (Figure 3b). The devices show broad photoresponses in the visible region (up to ~750 nm), in accordance with the low bandgap of P4TNTz-2F (~1.6 eV). The EQE spec-tra of the devices with and without the fullerene-SAM-treated interlayer are nearly identical; however, the EQE is larger for the device with the fullerene-SAM-treated in-terlayer than for the device without the SAM treatment, as can be expected from the higher *J*_SC_ of the fullerene-SAM-treated device. The *J*_SC_ calculated from the EQE data (*J*_SC,EQE_) agrees well with the measured *J*_SC_ of the devices.

**Figure 4:**
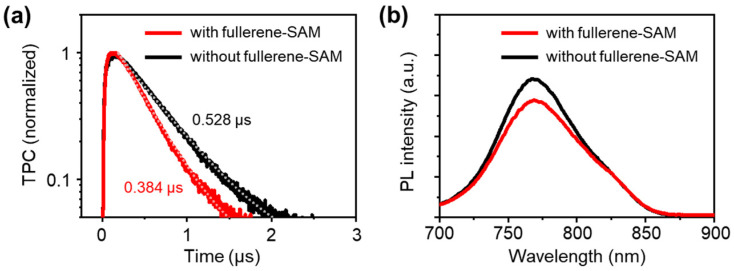
Characterization of changes in interfacial properties by fullerene-SAM treatment. (**a**) TPC decay profiles of solar cell devices. Solid lines: measured data points. Dotted lines: fitted lines (mono-exponential decay). (**b**) PL spectra of blend films on ZnO interlayer with and without SAM treatment. Excitation wavelength: 650 nm.

The origin of the increased photocurrent density due to the fullerene-SAM treatment was investigated. First, TPC measurements were performed to quantitatively assess the time required to extract photogenerated electrons from the organic photoactive layer to the electrodes. In the measurements, the solar cell devices were excited by a 3 ns laser pulse (532 nm) to generate a small amount of perturbed photogenerated electrons and holes in the devices under short-circuit conditions. Furthermore, the corresponding photoresponse was monitored as a function of time on the microsecond timescale, where the transient signal decayed as the photogenerated charge carriers were extracted by the electrodes. Thus, by measuring the characteristic decay time of the transient signal, it was possible to quantitatively compare how the electrodes efficiently extract charge carriers [15,16]. Further details of the TPC measurements can be found elsewhere [15,16]. The TPC measurements for the fullerene-SAM-treated and nontreated devices clearly show different decay rates for the photocurrent transients (Figure 4a). The decays are well-fitted by mono-exponential decay functions, giving decay constants of 0.384 µs and 0.528 µs for the fullerene-SAM-treated device and nontreated device, respectively. Considering that the structures of the devices are identical, except for the cathode interlayers (NPC70-OH/ZnO or ZnO), the difference in the TPC decay constants could be attributed to the difference in the electron-extracting ability of the cathode interlayers; the NPC70-OH SAM treatment enabled improved electron extraction from the organic photoactive layer. The improved electron extraction induced by the SAM could, in turn, reduce the series resistance (*R*_s_) of the device, where *R*_s_ = 4.52 Ω cm^2^ for the fullerene-SAM-treated device, compared with 5.12 Ω cm^2^ for the nontreated device (Table 2), contributing to the larger photocurrent density of the SAM-treated device.

In addition to fast electron extraction, the NPC70-OH SAM was expected to reduce the undesired contact between the ZnO interlayer and donor polymer P4TNTz-2F by covering the ZnO surface with C70 fullerenes. Reducing such undesired contacts is important for minimizing charge recombination loss at the organic–electrode interface (loss of photogenerated holes at the cathode interface in the case of the present work) and improving the device performance [15,18]. To determine the efficacy of the NPC70-OH SAM treatment for reducing the undesired contacts, PL quenching of the P4TNTz-2F:PC_71_BM blend film on ZnO due to the introduction of the NPC70-OH SAM was examined. If the fullerene-SAM covering the ZnO surface reduced direct contact between ZnO and P4TNTz-2F, the fullerene–P4TNTz-2F interfacial area would increase, leading to greater PL quenching of P4TNTz-2F. Comparison of the PL spectra of the P4TNTz-2F:PC_71_BM blend films (excited at 650 nm, where the light absorption of P4TNTz-2F predominates) on the ZnO or NPC70-OH/ZnO surfaces (Figure 4b) shows lower PL for P4TNTz-2F (centered at ~770 nm) in the blend film on the NPC70-OH/ZnO surface than that on the ZnO surface. This difference confirms the effectiveness of the SAM treatment in preventing undesired contacts and the consequent loss of charge carriers.

**Figure 5:**
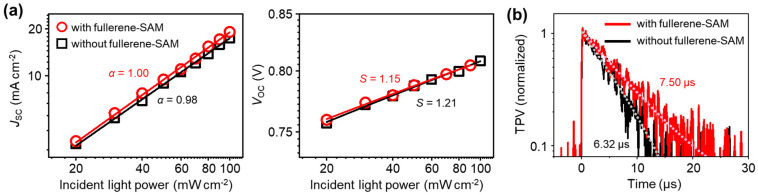
Characterization of charge recombination within solar cells. (**a**) Light power dependence of *J*_SC_ (left panel) and *V*_OC_ (right panel). Symbols: measured data points. Solid lines: lines fitted to equations given in the text. (**b**) TPV decay profiles. Solid lines: measured data points. Dotted lines: fitted lines (mono-exponential decay).

The effect of treating the ZnO interlayer with the NPC70-OH SAM on the charge recombination loss within the organic solar cells was also investigated. The degree of bimolecular charge recombination is quantified by measuring the dependence of the photocurrent density on the power of incident light (Figure 5a, left panel). The photocurrent density under short-circuit conditions is plotted as a function of the incident light power and fitted by the following equation: *J*_SC_~*P^α^*, where *P* is the light power, and the exponent *α* represents the degree of bimolecular charge recombination [42,43,44]. A value close to unity indicates negligible recombination, whereas smaller values indicate a greater degree of recombination [42,43,44]. The fitting yields *α* = 1.00 and 0.98 for the SAM-treated and nontreated devices, respectively, suggesting that the bimolecular recombination is less severe in the SAM-treated device than the nontreated device. The reduced bimolecular recombination in the SAM-treated device is attributed to improved electron extraction and less charge recombination at the ZnO/P4TNTz-2F contact. The dependence of *V*_OC_ on the light intensity is also measured (Figure 5a, right panel). The *V*_OC_ of an organic solar cell linearly depends on the natural logarithm of the light power according to the following equation: *V*_OC_~*S*(*kT*/*q*)ln *P*, where *k* is the Boltzmann’s constant, *q* is the elementary charge, *T* is the temperature, and *S* is a coefficient that represents the degree of trap-assisted recombination [45,46]. When the trap-assisted recombination within the device is negligible, *S* is close to unity, whereas *S* becomes greater as the trap-assisted recombination becomes more significant [45,46]. The slope of the *V*_OC_ vs. ln *P* plot is almost the same for the fullerene-SAM-treated and nontreated devices but is slightly smaller for the SAM-treated device (*S* = 1.15 and 1.21 for the device with and without SAM treatment, respectively). The similar *S* values suggest that the densities of the trap states in the two devices are nearly the same. This result is consistent with the similar microstructures of the blend films on the NPC70-OH/ZnO and ZnO surfaces. The slightly smaller *S* of the SAM-treated device plausibly originates from the passivation of the surface defects of the ZnO interlayer by SAM treatment [14,39].

The reduced bimolecular recombination in the SAM-treated device compared with that in the untreated device was further confirmed by TPV measurements. TPC measurements monitor the decay of the transient signal of laser pulse-induced photogenerated charges during their extraction through the electrodes, whereas TPV measurements monitor the decay of the transient signal by the bimolecular recombination of the photogenerated charges. Therefore, TPV measurements are generally performed on the devices under open-circuit conditions to prevent the extraction of charge carriers through the electrodes [15,16]. The decay time constant from TPV analysis indicates the lifetime of the charge carriers within the device, where a long charge carrier lifetime indicates a low degree of charge recombination within the device [15,16,47,48]. The TPV decays of the SAM-treated device and untreated device fit well to mono-exponential decay functions, where the decay constants are 7.50 µs for the SAM-treated device and 6.32 µs for the untreated device (Figure 5b). This difference indicates a lower degree of bimolecular recombination within the SAM-treated device, which is consistent with the light power dependence of *J*_SC_ (Figure 5a).

The above results demonstrate the effectiveness of the fullerene-SAM treatment of the ZnO interlayer for improving electron extraction and reducing undesirable ZnO/P4TNTz-2F contacts and defects at the ZnO surface. This leads to a decrease in the series resistance of the device and charge carrier losses during device operation and consequently improves the device performance.

## 4. Conclusions

In summary, we demonstrated that the formation of a fullerene-based SAM on the surface of the cathode interlayer is a simple and effective method of achieving efficient electron transfer at the organic–cathode interlayer interface. The SAM treatment provided the opportunity of fine-tuning the interfacial properties of the cathode interlayer. It facilitated electron extraction through the cathode and reduced charge carrier loss during device operation. Additionally, in the case of organic solar cells, the introduction of the fullerene-SAM led not only to the prevention of undesired cathode interlayer–donor polymer contact but also to the passivation of defect states at the cathode interface, resulting in a decrease in bimolecular recombination loss within the devices. Accordingly, the photocurrent density and PCE of the device showed meaningful improvements by the SAM treatment. This work highlighted the importance of optimizing the interfacial properties of organic semiconductor–electrode interfaces in organic optoelectronic devices and provided insights into how the interfacial properties are controlled by the introduction of SAMs at the interface and how these properties affect the device performance. Further systematic comparative investigations on other various types of SAMs will add depth to this understanding, which would be covered in our future studies.

## Figures and Tables

**Table 1:** Previous reports on performance enhancement of organic solar cells by SAM.

Name of SAM	Active Material	Best PCE (w/o SAM) (%)	Best PCE (w/SAM) (%)	Ref
C_n_F_4_PAPTSi	PTB7:PC_71_BM	1.85	7.62	[22]
BBC	P3HT:PC_61_BM	2.51	2.90	[23]
C3	PBDB-T:ITIC	10.03	10.90	[27]
Cz-BA	P3HT:PC_61_BM	2.49	2.88	[28]
MUA	P3HT:PC_61_BM	3.2	4.6	[29]
C-PCBOD	P3HT:PC_61_BM	3.57	4.50	[32]
Bis-*p*-EHO-PCBA	PTBT:PC_61_BM	3.28	5.13	[33]
C_60_	P3HT:PC_61_BM	3.74	4.54	[34]
C_60_-COOH	P3HT:PC_61_BM	3.47	4.40	[35]
PEEMC	PTB7:PC_71_BM	6.6	7.5	[36]
Open-PCBM	PIDTT-DFBT:PC_71_BM	6.24	7.67	[38]
NPC70-OH	*p*-DTS(FBTTh_2_)_2_:PC_71_BM	8.30	9.14	[39]

**Table 2:** Photovoltaic parameters of devices with and without fullerene-SAM treatment.

Cathode Interlayer	PCE (%)	*J*_SC_ (mA cm^−2^)	*V*_OC_ (V)	FF	*R*_s_ (Ω cm^2^)	*J*_SC,EQE_ (mA cm^−2^)
NPC70-OH SAM/ZnO	10.8 (10.4 ± 0.184) *	20.6 (19.7 ± 0.516) *	0.81 (0.82 ± 0.008) *	0.650 (0.650 ± 0.018) *	4.52	20.2
ZnO	9.83 (9.38 ± 0.275) *	18.9 (18.2 ± 0.852) *	0.81 (0.81 ± 0.005) *	0.642 (0.635 ± 0.033) *	5.12	18.4

* Averaged over 10 devices.

## Data Availability

The data presented in this study are available on request from the corresponding author.
